# Self-Contained Induction of Neurons from Human Embryonic Stem Cells

**DOI:** 10.1371/journal.pone.0006318

**Published:** 2009-07-21

**Authors:** Tsuyoshi Okuno, Takashi Nakayama, Nae Konishi, Hideo Michibata, Koji Wakimoto, Yutaka Suzuki, Shinji Nito, Toshio Inaba, Imaharu Nakano, Shin-ichi Muramatsu, Makoto Takano, Yasushi Kondo, Nobuo Inoue

**Affiliations:** 1 Advanced Medical Research Laboratory, Mitsubishi Tanabe Pharma Corporation, Osaka, Japan; 2 Department of Advanced Pathobiology, Graduate School of Life and Environmental Sciences, Osaka Prefecture University, Osaka, Japan; 3 Department of Biochemistry, Yokohama City University School of Medicine, Yokohama, Japan; 4 Division of Neurology, Department of Medicine, Jichi Medical University, Tochigi, Japan; 5 Department of Physiology, Jichi Medical University, Tochigi, Japan; 6 Laboratory of Regenerative Neurosciences, Graduate School of Human Health Sciences, Tokyo Metropolitan University, Tokyo, Japan; University of Sydney, Australia

## Abstract

**Background:**

Neurons and glial cells can be efficiently induced from mouse embryonic stem (ES) cells in a conditioned medium collected from rat primary-cultured astrocytes (P-ACM). However, the use of rodent primary cells for clinical applications may be hampered by limited supply and risk of contamination with xeno-proteins.

**Methodology/Principal Findings:**

We have developed an alternative method for unimpeded production of human neurons under xeno-free conditions. Initially, neural stem cells in sphere-like clusters were induced from human ES (hES) cells after being cultured in P-ACM under free-floating conditions. The resultant neural stem cells could circumferentially proliferate under subsequent adhesive culture, and selectively differentiate into neurons or astrocytes by changing the medium to P-ACM or G5, respectively. These hES cell-derived neurons and astrocytes could procure functions similar to those of primary cells. Interestingly, a conditioned medium obtained from the hES cell-derived astrocytes (ES-ACM) could successfully be used to substitute P-ACM for induction of neurons. Neurons made by this method could survive in mice brain after xeno-transplantation.

**Conclusion/Significance:**

By inducing astrocytes from hES cells in a chemically defined medium, we could produce human neurons without the use of P-ACM. This self-serving method provides an unlimited source of human neural cells and may facilitate clinical applications of hES cells for neurological diseases.

## Introduction

Embryonic stem (ES) cells, derived from the inner cell mass of blastocysts, are pluripotent cells that can differentiate into a variety of cell types including neural cells [1], [2]. Among the various basic and clinical applications for ES cells, cell transplantation therapy for central nervous diseases is of particular interest because differentiated neurons do not proliferate, and a relatively large number of donor cells are necessary to replace diseased neurons. Several methods have been developed to prepare neural cells from ES cells. Neurons can be obtained indirectly from ES cells via ectodermal cells in embryoid bodies, which are formed from dissociated ES cells, either by induction with retinoic acid or selection [3], [4]. Alternatively, neural stem cells and neurons can be directly differentiated from ES cells without forming embryoid bodies by culturing ES cells on mouse-cultured stroma cells (PA-6) [5], or under chemically defined low-density culture conditions [6]. All of these procedures, however, are time consuming and require highly complicated processes to generate many neurons. In addition, their practicality is limited by the possible teratogenicity caused by culture factors, such as retinoic acid, of differentiated cells. We have previously reported an efficient method to prepare transplantable neural cells from mouse ES cells using a conditioned medium collected from rat primary-cultured astrocytes (P-ACM) [7]–[9]. In this study, we applied this method to human ES (hES) cells for induction of neurons and astrocytes. Once the astrocytes were derived from hES cells, they could be substituted for primary astrocytes that induce neurons, thus achieving xeno-free production of neurons.

## Results

### Neural cell differentiation from hES

**Figure 1:**
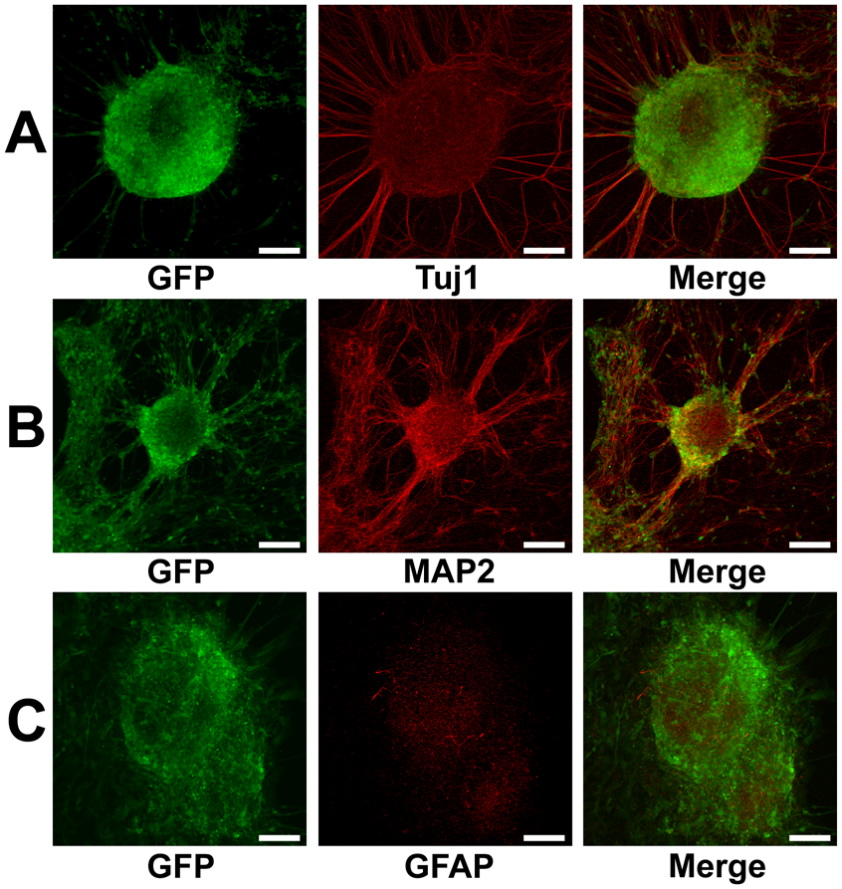
Differentiation of hES cells into neurons in P-ACM. Floating spheres composed of neural stem cells and undifferentiated cells grown for 12 days were plated on an adhesive substrate and cultured for 14 days in P-ACM. Expression of hrGFP (green), Tuj1 (A, red), MAP2 (B, red), and GFAP (C, red) staining of the many neural and few glial cells derived from hES cells. Bar = 100 µm.

Four hES cell-lines stably expressing humanized renilla green fluorescent protein (hrGFP) were obtained. These hES cell-lines were kept in undifferentiated state with positive stem cell markers, such as alkaline phosphatase, Oct-4, and SSEA-4. When cultured in P-ACM containing fibroblast growth factor-2 (FGF-2) under free-floating conditions, colonies of undifferentiated hES cells gave rise to floating spheres composed of neural stem cells and undifferentiated cells, which gradually increased in size during the culture. After 12 days of culture, the spheres were plated onto a poly-L-Lysine/Laminin coated dish and cultivated in neural stem cell medium (NSCM) containing high concentrations of FGF-2 and epidermal growth factor (EGF). Within 24 h, the spheres attached onto the substrate and formed circular clusters of cells. Many of these cells subsequently migrated to the surrounding areas and covered the growth surface of the dish in circular monolayers. After replacing NSCM by P-ACM and culture for 14 days, the spheres differentiated into neurons (Figures 1A, B) and few astrocytes (Figures 1C). These were identified by the neuronal marker tubulin β III isoform (Tuj1) and the astrocytic marker glial fibrillary acidic protein (GFAP).

### Selective differentiation of hES cells into neurons and astrocytes

**Figure 2:**
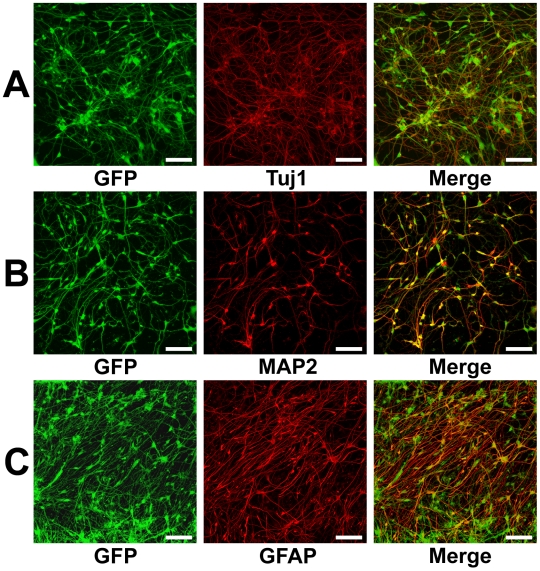
Selective induction of hES cells into neurons and astrocytes. (A, B): Neural stem cells that had migrated from floating spheres in NSCM were subcultured onto a PLL coated plate and cultured for 14 days in P-ACM. Immunostaining with antibody to Tuj1 and MAP2 showed that the subcultured neural stem cells had differentiated into neurons. Expression of hrGFP (green), (A) Tuj1 (red), and (B) MAP2 (red) staining profiles. (C): Neural stem cells were cultured for 14 days after removal of the core of spheres with a glass pipette and change of medium to G5 medium. The proliferated cells were subcultured onto PLL/LAM coated plate and cultured for 14 days in G5 medium. Immunostaining with antibody to GFAP showed that the subcultured cells had differentiated into astrocytes. Expression of hrGFP (green) and GFAP (red) staining profiles. Bar = 100 µm.

By culture of the spheres in NSCM, neural stem cells were able to migrate from the attached spheres to the surrounding area and subsequently form a circular cluster. NSCM containing FGF-2 and EGF promotes neural stem cells proliferation, while repressing their differentiation into any type of neural cells. After removing the core of the attached spheres mechanically, the remaining neural stem cells could proliferate in NSCM and selectively differentiate into neurons and astrocytes by subculture in an appropriate medium. To differentiate into neurons, neural stem cells were subcultured using 0.05% Trypsin/EDTA in P-ACM for 14 days (Figures 2A, B). After these 14 days of subculture, a large number of cells expressed Tuj1 (84.0±5.1%, *n* = 3). On the other hand, to differentiate into astrocytes, neural stem cells were subcultured in G5 medium for 14 days (Figure 2C). After this subculture, a large number of cells expressed GFAP (75.0±1.2%, *n* = 3). The removed core of the attached spheres could, like the first spheres, be used repeatedly (about twenty times) as seed for neural stem cells.

### Xeno-free induction of astrocytes using a chemically defined medium

**Figure 3:**
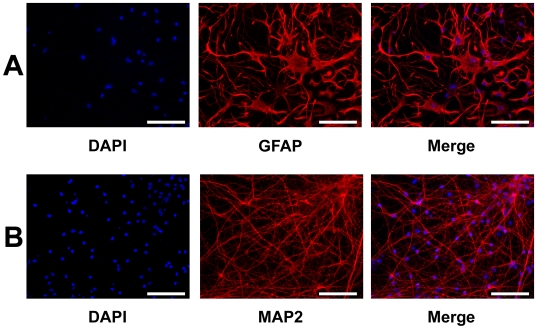
Differentiation of hES cells into astrocytes in a chemically defined medium. (A): Neural stem cells induced by N2 medium were cultured for 14 days after removal of the core of spheres with a glass pipette and change of medium to G5 medium. The proliferated cells were subcultured onto a PLL/LAM coated plate and cultured for 14 days in G5 medium. Immunostaining with antibody to GFAP showed that most of the subcultured cells had differentiated into astrocytes. DAPI (blue) and GFAP (green) staining profiles. Neural stem cells induced by xeno-free ES-ACM were subcultured onto PLL coated plate and cultured for 6 weeks in ES-ACM. (B): Immunostaining with antibody to MAP2 showed that the subcultured NSCs had differentiated into mature neurons. DAPI (blue) and MAP2 (red) staining profiles. Bar = 100 µm.

For collection of xeno-free astrocytes derived from hES cells, we used a chemically defined N2 medium for neural induction. When cultured in N2 medium containing FGF-2 and EGF under free-floating conditions, colonies of undifferentiated hES cells gave rise to floating spheres. As N2 was less efficient than P-ACM for obtaining astrocytes, we prepared hES cells in large scale (>10^8^ cells). After differentiation, millions of astrocytes were gained under xeno-free condition (Figure 3A).

### Neuronal induction of hES cell-derived astrocytes

**Figure 4:**
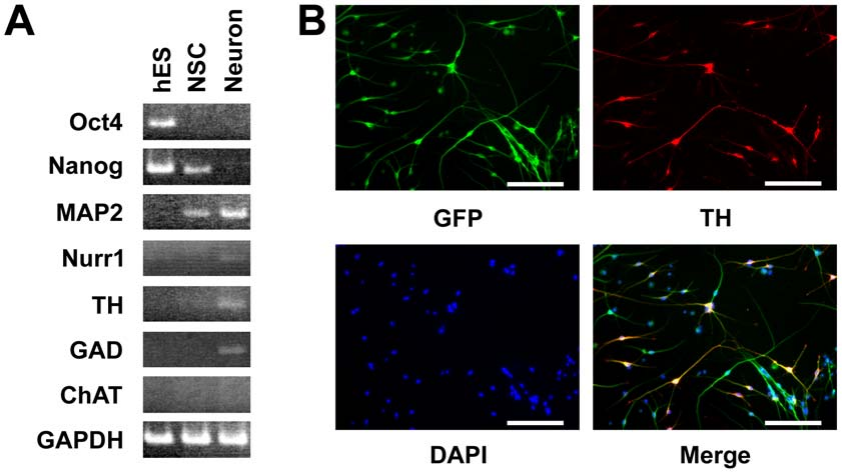
RT-PCR analysis and differentiation of hES cells into dopaminergic neurons in xeno-free ES-ACM. (A): RT-PCR analysis of hES cells, neural stem cells and mature neurons. RNA was isolated from clones of undifferentiated hES cells, from neural stem cells, and from mature neurons which had been cultured for 8 weeks in ES-ACM and analyzed for expression of marker genes. The expression levels of each gene were normalized to GAPDH gene expression level. hES, undifferentiated hES cells; NSC, neural stem cells; Neuron; mature neurons. (B): Differentiation of hES cells into dopaminergic neural cells in xeno-free ES-ACM. Neural stem cells induced by ES-ACM were subcultured onto PLL coated plate and cultured for 8 weeks in ES-ACM. Immunostaining with antibody TH and expression of hrGFP showed that the subcultured neural stem cells had differentiated into dopaminergic neurons. Expression of hrGFP (green), DAPI (blue) and TH (red) staining profiles. Bar = 100 µm.

Next, we investigated whether the astrocytes derived from hES cells can be substituted for primary astrocytes to differentiate hES cells into neural cells. A conditioned medium of hES cell-derived astrocytes was collected after two days culture and used as ES-ACM by adding an equal amount of N2 medium. As in the case of P-ACM, hES cells cultured in ES-ACM differentiated into neural cells via formation of spheres. After switching the cells from NSCM to ES-ACM, many cells had neuronal-like appearance with long neurites. By 6 weeks of culture in ES-ACM, most cells had neural morphology and expressed microtubule-associated protein 2 (MAP2) (Figure 3B). During our procedure for differentiating hES cells using ES-ACM, expression of several markers was analyzed by RT-PCR (Figure 4A). By 8 weeks culture in ES-ACM, some cells showed tyrosine hydroxylase (TH) -immunoreactivity (Figure 4B). Furthermore, when cultured in ES-ACM again, the cells could differentiate into astrocytes via formation of spheres. To induce differentiation of neural stem cells into astrocytes, the culture medium was changed from NSCM to G5 medium. After medium change, most neural stem cells had the appearance of typical astrocytes. By 2 weeks culture in G5 medium, the majority (82.4±1.8%, *n* = 3) of cells expressed GFAP, and few (<1%) expressed MAP2.

### Electrophysiological analysis of neurons differentiated from hES cells

**Figure 5:**
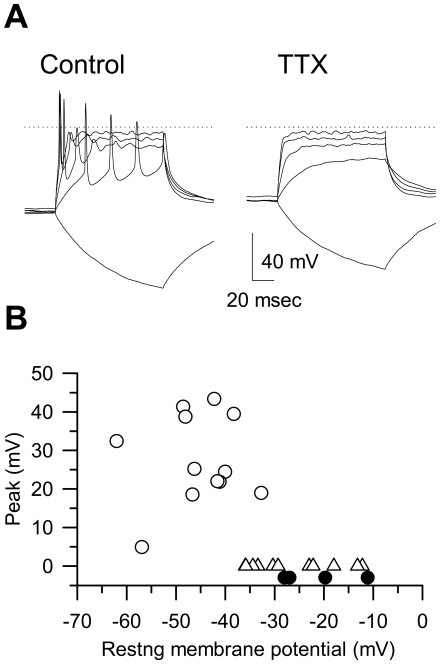
Electrophysiological properties of hES-derived neurons. (A): (Control) Action potentials elicited by depolarizing current injections (50 pA steps for 100 msec). The resting membrane potential was −62.0 mV. During the hyperpolarizing pulse (−50 pA), no ‘sug’ component was observed. (TTX) Membrane potentials recorded in the presence of 1 µM tetrodotoxin. In both panels, the dotted lines indicate 0 mV. (B): Summary of the resting membrane potentials and the peak amplitudes. Open circle; cells with action potentials. Open triangle; cells with action potentials only when the resting membrane potential was set to −70 mV. Closed circle; cells without membrane excitability.

For electrophysiological study, hES-derived neurons were cultured on coverslips for 4–6 weeks. The coverslips were transferred to a recording chamber before use. Neurons were selected based on their appearance (spherical shape with long neurites). The resting membrane potential of the neurons were −62.0∼−11.1 mV (*n* = 26). Among 26 cells examined, action potentials were elicited in 22 cells (Figure 5A, Control). Application of 1 µM tetrodotoxin (TTX) completely suppressed the overshoot (Figure 5A, right panel). The action potentials were evoked only when resting membrane potentials were set to −70 mV by current injection in 11 cells. The rest of the cells did not possess membrane excitability (*n* = 4 out of 26, closed circle in Figure 5B).

### Survival of hES cell-derived neurons in mice brain

**Figure 6:**
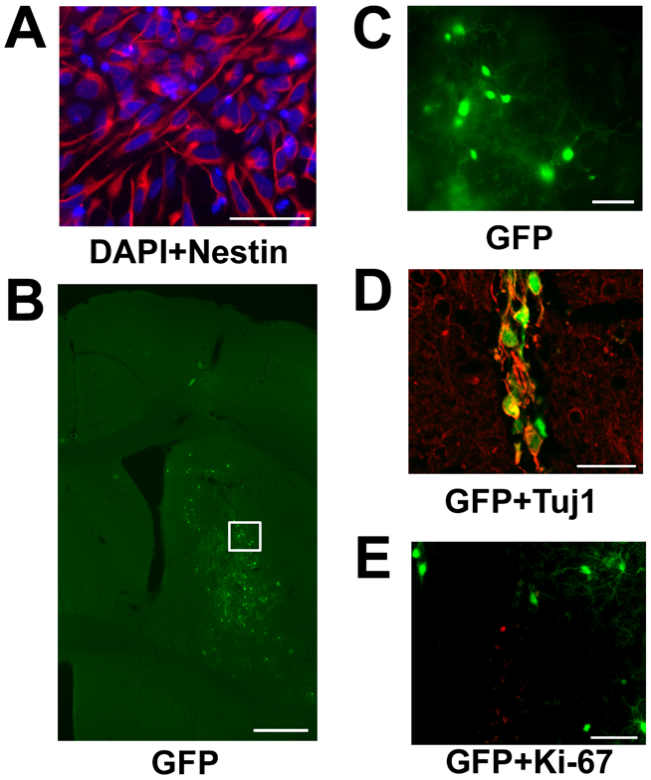
Survival of transplanted neural stem cells *in vivo*. (A): Most of the donor cells were confirmed to be Nestin-immunoreactve neural stem cells before transplantation. Anti-Nestin staining (green) and DAPI (blue). Bar = 50 µm. (B): Transplantation site. Grafted cells expressing hrGFP can be seen in the striatum. Bar = 500 µm. (C): High power magnification view of a white box in panel A. Some of the hrGFP-positive cells display a morphology similar to that of neurons. Bar = 50 µm. (D): Merged image of hrGFP expression (green) and immunostaining of anti-Tuj1 (red). Bar = 20 µm. (E): Merged image of hrGFP expression (green) and immunostaining of anti-Ki-67 (red). Bar = 50 µm.

To examine differentiation of hES cell-derived cells *in vivo*, we transplanted neural stem cells induced with ES-ACM into mice brain (*n* = 7). Before transplantation, the majority (85.1±5.1%, *n* = 4) of donor cells expressed Nestin, a marker for neural stem cell (Figure 6A), and no cells expressed octamer transcription factor-3 (Oct-3) and stage-specific embryonic antigen (SSEA-4), two makers for undifferentiated cells (data not shown). Four weeks after engraftment, many (2–3×10^2^) hrGFP-positive cells were recognized (Figure 6B, C). Some of these cells (<10%) were also Tuj1-immunoreactive (Figure 6D). In the vicinity of the grafts, few cells were immunoreactive against Ki-67, a marker for proliferation. However, none of these Ki-67-positive cells were positive for hrGFP (Figure 6E). Teratoma was not detected in any of the transplanted mice.

## Discussion

We have shown in this study that neurons and astrocytes can be produced efficiently from hES cells using a conditioned medium collected from either rat primary-cultured astrocytes or hES cell-derived astrocytes. Astrocytes derived from hES cells can be used for continuous generation of neurons. Although a number of media including serum-free media supplemented with various cytokines and/or growth factors have been developed [10], [11] to keep a long-term culture of neuronal cells, synthetic culture systems can usually maintain neural cells stable for only few weeks. Although a conditioned medium of primary-cultured astrocytes can be effective in culturing neurons for a longer period of time, the use of primary astrocytes may not be practical due to a number of limitations, including restricted availability of neural tissues as source of astrocytes, and extensive time and effort to obtain astrocytes from living tissues. Additionally, it is very difficult to maintain a stable culture of primary cells in a culture vessel, and subculture of these cells is limited within few passages.

The properties of primary-cultured astrocytes vary depending on the maturation stage of the living body and the region of the living tissue from which the astrocytes are derived. In addition, when astrocytes are obtained from a living body, contamination with cells other than the desired astrocytes is inevitable. Thus, it is difficult to prepare a stable astrocyte-conditioned medium having substantially uniform quality. With our method, on the other hand, ES-ACM can efficiently induce differentiation of hES cells into neural cells. Moreover, large amounts of ES-ACM can be produced stably and readily. ES-ACM, like P-ACM, can keep neuron cultures stable for more than eight weeks until mature neuronal phenotypes are apparent. In addition, completely xeno-free ES-ACM can be generated from immature hES cells by culture in chemically defined medium. With this completely xeno-free ES-ACM, xeno-free neurons and astrocytes can repeatedly be produced. In our transplantation experiment, donor cells did not express undifferentiated markers, such as Oct-3 and SSEA-4. In addition, only few Ki-67 positive cells found in the vicinity of the grafts were hrGFP-negative. These cells were unlikely to be derived from donor cells. It is important to exclude tumorigenicity of neuronal cells derived with this xeno-free method in future study for application to the cell therapy. Further studies are necessary to identify the specific molecules that induce neural cells in P- and ES-ACMs.

Recently, two methods adopting human tissue-derived cells have been reported as appropriate for clinical applications. One is an improved stromal cell-induced method that uses an amniotic membrane matrix [12]. The other uses telomerase-immortalized midbrain astrocytes [13]. Although both methods are xeno-free, they still need primary human tissues. On the other hand, with our method, neural cells can be induced from ES cells themselves. This self-serving method can supply donor cells consistently and may have an advantage for clinical applications.

## Materials and Methods

### ES cell culture

All experiments using hES cells were performed in conformity with “The Guidelines for Derivation and Utilization of Human Embryonic Stem Cells” of the Japanese government after approval by the institutional review board of Mitsubishi Tanabe Pharma Corporation. Two hES cell lines, SA002 and SA181, were obtained from Cellartis AB (Goteborg, Sweden) [14] and maintained on a mitotically inactivated mouse embryonic fibroblast feeder layer in a culture medium (vitroHES, Vitrolife AB, Goteborg, Sweden), supplemented with 4 ng/ml FGF-2 (Invitrogen, Carlsbad, CA). For passaging, the hES cells were treated with collagenase type IV (200 U/ml; Invitrogen) for 5 min, gently scraped from the culture dish, and then split 1∶2–1∶4 onto a feeder layer of mouse embryonic fibroblasts inactivated with 10 µg/ml mitomycin C.

### Electroporation

All recombinant DNA experiments conformed to National Institute of Health (NIH) guidelines. First, a pGFP plasmid, in which hrGFP (Stratagene, La Jolla, CA) was expressed under the control of a CAG promoter (a gift from J. Miyazaki) [15] was constructed. Ten micrograms of the linearized plasmid was then electroporated into a suspension of hES cells (10^7^ cells) in 0.8 mL of PBS using a Gene Pulser (500 µF, 250 V, Bio-Rad, Hercules, CA). The cells were next incubated on ice for 10 minutes, plated, and allowed to recover for 24 hours before selection with G418 (200 µg/mL). The cells were daily fed with the culture medium containing G418 for 12 days, after which the resulting ten G418-resistant ES colonies showing strong hrGFP expression were individually picked and propagated. To analyze stem cell markers, alkaline phosphatase activity and cell surface markers were detected using an ES cell characterization kit (Chemicon, Temecula).

### hES cell differentiation

Whole colonies of undifferentiated hES cells, 800–1000 µm in diameter, were picked up from the feeder layer using a glass capillary and transferred into non-adhesive bacteriological dishes each containing P-ACM supplemented with 20 ng/ml FGF-2 (R&D Systems Inc., Minneapolis). P-ACM was prepared as described previously [7]. The colonies were then cultured for 12 days, giving rise to spheres, which were next plated onto poly-L-Lysine/Laminin (Sigma-Aldrich, St. Louis) coated dishes and cultivated for seven days in NSCM (Neurobasal medium supplied with B27 supplement, both from Invitrogen, 20 ng/ml FGF-2, and 20 ng/ml recombinant EGF [R&D systems]). At this stage, the spheres gave rise to circular clusters of cells, many of which migrated from the clusters to the surrounding areas. After replacing the NSCM with P-ACM and culture for 14 days, the spheres differentiated into neurons and few astrocytes. To obtain more and purer neurons, the centers of the clusters containing undifferentiated ES cells were removed with a glass capillary, and the rest of the clusters were cultured for seven days in NSCM. Neuronal differentiation was then induced by subculture of neural stem cells using 0.05% Trypsin/EDTA in P-ACM for 14 days. To induce astrocytic differentiation, the neural stem cells were subcultured in G5 medium (Neurobasal medium supplemented with G5 supplement, both from Invitrogen, 10 ng/ml FGF-2, and 20 ng/ml EGF) for 14 days.

To induce astrocytic differentiation under xeno-free conditions, the colonies of hES cells were transferred into non-adhesive bacteriological dishes each containing N2 medium (Neurobasal medium supplied with N2 supplement, Invitrogen) supplemented with 20 ng/ml of FGF-2 and EGF. After the colonies were cultured for 12 days, few of them gave rise to spheres containing neural stem cells, which were subsequently plated onto poly-L-Lysine/Laminin in G5 medium. Centers of the spheres containing undifferentiated hES cells were removed with a glass capillary, and the rest of the clusters were cultured for seven days in G5 medium. By repeating over this cycle eight times, the spheres were purified to obtain pure neural stem cells. These neural stem cells were subcultured in G5 medium for 14 days to induce astrocytes. For collection of ES-ACM, hES cells derived-astrocytes were cultured in N2 medium for two days.

### Immunostaining analysis

hES cells cultured in a 24-well plate were fixed in 4% paraformaldehyde in phosphate-buffered saline (PBS). Immunocytochemistry was performed using standard protocols and antibodies directed against Tuj1 (monoclonal 1∶1000), MAP2 (monoclonal, 1∶1000), GFAP (polyclonal 1∶500), Oct-4 (monoclonal 1∶500), SSEA-4 (monoclonal 1∶400), Nestin (monoclonal 1∶1000) (all from Chemicon), and TH (monoclonal, 1∶400) (Acris Antibodies, Hiddenhausen, Germany). Alexa Fluor 594-labeled (Molecular Probes, Eugene, OR) and Cy3-labeled (GE healthcare, Uppsala, Sweden) secondary antibodies were used for visualization. 4′, 6-diamidino-2-phenylindole (DAPI, Kirkegaard Perry Laboratories, Gaithersburg) was used for nuclei staining.

Cell density of neural lineages (neurons and astrocytes) was determined by counting the numbers of DAPI, Tuj^+^ and GFAP^+^ cells per field at a magnification of 200 times using an inverted microscope. Five visual fields were randomly selected and counted for each sample. Numbers presented in figures represent the average percentage and SEM of positive cells over DAPI from three samples per each examination.

### RT-PCR analysis

Total RNA was extracted from undifferentiated hES cells, neural stem cells, and neuronal cells using QIAshredder (QIAGEN, Hilden, Germany) and RNeasy Plus Mini kit (QIAGEN). Reverse transcription was carried out using random hexamers at 37°C for 60 minutes according to the manufacturer's instruction for First-Strand cDNA Synthesis Kit (GE Healthcare UK Ltd., Buckinghamshire, UK). PCR was carried out for 30 cycles using the specific primer sets. The reaction cycle was set at 95°C for 30 seconds, 55°C for 30 seconds, and 72°C for 30 seconds. The amplified fragments were subjected to electrophoresis in a 2% agarose gel, which was subsequently stained with ethidium bromide and photographed. The primers used are as follows: glyceraldelyde-3-phosphatedehydrogenase (GAPDH), ACCACAGTCCATGCCATCAC and TCCACCACCCTGTTGCTGTA; Oct4, CGTTCTCTTTGGAAAGGTGTTC and ACACTCGGACCACGTCTTTC; Nanog, AAGACAAGGTCCCGGTCAAG and CCTAGTGGTCTGCTGTATTAC; MAP2, CTTTCCGTTCATCTGCCATT and GCATATGCGCTGATTCTTCA; Nurr1, GCTAAACAAAACTTGCATGC and CTCATATCATGTGCCATACTAG; TH, GAGTACACCGCCGAGGAGATTG and GCGGATATACTGGGTGCACTGG; choline acetyltransferase (ChAT), ATGGGGCTGAGGACAGCGAAG and AAGTGTCGCATGCACTGCAGG; glutamic acid decarboxylase (GAD), ATTCTTGAAGCCAAACAG and TAGCTTTTCCCGTCGTTG.

### Electrophysiology

The action potential was recorded using current clamp mode of Axopatch200B amplifier and Digidata 1320 interface (Axon, CA, USA). Physiological bathing solution contained (in mM); 140 NaCl, 5.4 KCl, 0.33 NaH_2_PO_4_, 0.5 MgCl_2_, 1.8 CaCl_2_, 5 HEPES (pH = 7.4 with NaOH). Standard high K^+^ pipette solution contained; 110 Aspartic acid, 30 KCl, 5 MgATP, 5 Na_2_ creatine phosphate, 0.1 Na_2_GTP, 2 EGTA, 10 HEPES (pH = 7.2 with KOH). Electrode resistance was 8∼6 MOhm. All experiments were carried out at 33–35°C.

### Transplantation Experiment

Neural stem cells derived from hES cells using ES-ACM were implanted into the mouse striatum. All animal experimental protocols were approved by the Animal Ethics Committee of Mitsubishi Tanabe Pharma Corporation. 8-week-old C57BL/6 Cr Slc mice (SLC, Shizuoka, Japan) were anesthetized with pentobarbital and fixed on a stereotactic device (Narishige, Tokyo, Japan). By using a glass pipette with an inner diameter of 100 µm, 1×10^5^ cells/5 µl were slowly (0.3 µl/min) injected into the striatum (AP±0 mm, ML +2.0 mm, DV −3.0 mm from bregma) of an adult male mouse. Four weeks after the transplantation, the recipient mouse was anesthetized with pentobarbital and perfused with ice-cold 4% paraformaldehyde in PBS. The brains of each mouse were postfixed in the same solution, cryoprotected with 30% sucrose in PBS for 48 h, and frozen. Coronal sections (thickness 40 µm) were cut on a microtome with freezing unit, collected in PBS (pH 7.4), and divided into series. Brain sections were incubated overnight with primary antibodies at 4°C. The primary antibodies used for immunohistochemistry ware mouse anti-Tuj1 (1∶800, Covance, USA) and rabbit anti-Ki67 (1∶25, abcam, UK). For detection of the primary antibodies, Alexa Fluor 594 goat anti-mouse IgG (1∶1000; Molecular Probes) and Alexa Fluor 594 goat anti-rabbit IgG (1∶1000; Molecular Probes) were incubated with the samples. Immunoreactivity was assessed and viewed under confocal laser scanning microscopy (FV10i; Olympus, Tokyo).
